# Development of a Low-Shrinkage-Lightweight Engineered Cementitious Composite Based on Heavily Doped Zeolites

**DOI:** 10.3390/polym15163474

**Published:** 2023-08-19

**Authors:** Yue Wang, Rongxin Guo, Dian Guan, Zhiqiang Luo, Ziqi Zhang, Runsheng Lin

**Affiliations:** 1 Faculty of Civil Engineering and Mechanics, Kunming University of Science and Technology, Kunming 650500, China; 20212210033@stu.kust.edu.cn (Y.W.); 17608897404@163.com (D.G.); lzq955882023@163.com (Z.L.); 15237228950@163.com (Z.Z.); linrunsheng@kust.edu.cn (R.L.); 2 Yunnan Key Laboratory of Disaster Reduction in Civil Engineering, Kunming 650500, China; 3 International Joint Laboratory for Green Construction and Intelligent Maintenance of Yunnan Province, Kunming 650500, China

**Keywords:** LECC, zeolite, polyethylene fiber, ductility, autogenous shrinkage, microanalysis

## Abstract

In recent years, there has been a growing utilization of lightweight engineered cementitious composites (LECC) for the reinforcement and restoration of contemporary building structures. This study focuses on the incorporation of zeolite, serving as an internal reservoir for moisture maintenance, and examines its impact on various performance indicators, including apparent density, compressive strength, tensile strength, and autogenous shrinkage. Additionally, the influence of zeolite on the tensile and ductile properties of LECC is elucidated with the aid of scanning electron microscopy (SEM). The findings reveal that the addition of zeolite enables the preservation of excellent mechanical properties of LECC while further reducing its density. Notably, the introduction of a substantial amount of zeolite leads to a decrease in matrix density, average crack width, and ultimate tensile strain. The ultimate tensile strain exceeds 8% to reach 8.1%, while the decrease in compressive and tensile strengths is marginal. Zeolite’s internal curing capability facilitates the complete hydration of unhydrated cement, concurrently alleviating the autogenous shrinkage of LECC. Consequently, the durability and reliability of the material are enhanced. The ability of zeolite, with its porous framework structure, to significantly improve the ultimate tensile strain of the matrix can be attributed to the amplified occurrence of active defects and a shift in the pull-out mode of PE fibers from “pull-out” to “pull-through”. This study presents a promising alternative material in the field of engineering, holding potential for diverse building and infrastructure projects, as it enhances their durability and reliability.

## 1. Introduction

Traditional building materials, such as reinforced concrete, impose significant deadweight loads on building structures due to their high density and weight. Moreover, with the escalating concerns about global warming and energy consumption [1], there is an urgent need for the construction industry to develop new materials that reduce structural loads and energy consumption and enable the design of environmentally friendly and lightweight precast concrete components [2]. In this context, lightweight engineered cementitious composites (LECCs) have emerged as a promising solution [3,4]. They possess lower thermal conductivity, owing to the inclusion of lightweight aggregates or foaming agents, and exhibit excellent thermal insulation properties, effectively reducing heat conduction and energy loss within buildings [5,6]. This advantage overcomes the challenges faced by traditional building materials in terms of weight and energy consumption, making LECC a highly precious material for sustainable construction and engineering.

In recent years, LECC has witnessed significant advancements, particularly in terms of strength and density. Huang et al. [7] discovered that the incorporation of hollow beads as additives effectively reduces the density of ECC, enhances tensile ductility, reduces crack width, and decreases thermal conductivity. These improvements contribute to energy savings and environmental protection in building structures. Zhang et al. [8] replaced traditional polyvinyl alcohol (PVA) fibers with polyethylene (PE) fibers and successfully developed LECC with a density ranging from 1400 to 1700 kg/m^3^. This LECC exhibited high tensile and compressive strengths of 7–8 MPa and 40–70 MPa, respectively, and could withstand tensile strains of up to 8%. Fu et al. [9] introduced nanoscale additives, such as nano-silica (NS) and silica powder (SF), which effectively enhanced the mechanical properties of LECC by increasing strength and durability.

While previous research on LECC primarily focused on mechanical properties and durability, it has become evident that LECC exhibits greater shrinkage compared to ordinary concrete, including substantial autogenous shrinkage. Despite the crucial role of shrinkage properties in material stability and structural durability, current research has not adequately addressed the shrinkage issue of LECC, necessitating further comprehensive explorations and investigations. To mitigate the shrinkage of cementitious composites, internal curing using highly absorbent polymers (SAPs) and lightweight aggregates (LWAs) represents a common approach. SAPs facilitate long-term internal curing of cementitious materials while reducing their shrinkage. However, the addition of SAPs tends to introduce additional porosity, which reduces concrete strength [10,11,12]. Another effective method to reduce shrinkage involves incorporating pre-wetted lightweight aggregates [13,14,15,16] into cementitious composites, serving as internal reservoirs to provide a source of water during curing. Zeolite has gained attention in recent years as a lightweight aggregate for the interior maintenance of ECC [17,18,19]. Natural zeolite, with its porous framework structure, can adsorb water up to 30% of its own weight [20] while reducing the density of cementitious materials. In this study, zeolite is utilized as a lightweight aggregate in LECC to modulate shrinkage, leveraging its inherent advantages. The porous structure of zeolite acts as a water reservoir, reducing shrinkage and facilitating internal maintenance to preserve the lightweight property of LECC. Thus, employing zeolite for shrinkage regulation provides a natural advantage in producing low-shrinkage LECC.

Based on the above analyses, the objective of this study was to develop LECCs with low shrinkage, low density, and high ductility. For this purpose, experiments were conducted using zeolite as a lightweight aggregate. This study included macro- and microscopic property evaluations such as apparent density, mechanical properties, and autogenous shrinkage properties. In addition, the effect of polyethylene fiber-matrix interfacial bonding on the tensile properties of LECC was observed using scanning electron microscopy (SEM), and the shrinkage regulation principle of LECC was explained by internal relative humidity measurements. The experimental results showed that the low specific gravity of calcined zeolite (1.08 g/cm^3^) enabled the LECC to maintain a low density while giving the matrix high ductility (8.1%). Secondly, as a shrinkage-regulating internal curing agent, zeolite can provide internal curing water to LECC, effectively mitigating its autogenous shrinkage.

## 2. Materials and Methods

### 2.1. Materials

**Figure 1:**
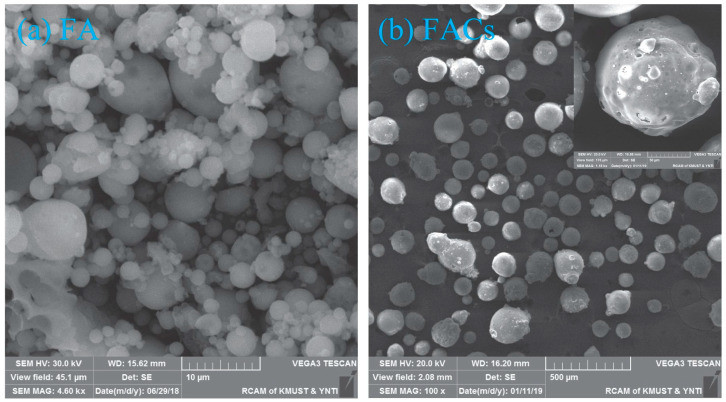
SEM images of (**a**) FA and (**b**) FACs.

**Figure 2:**
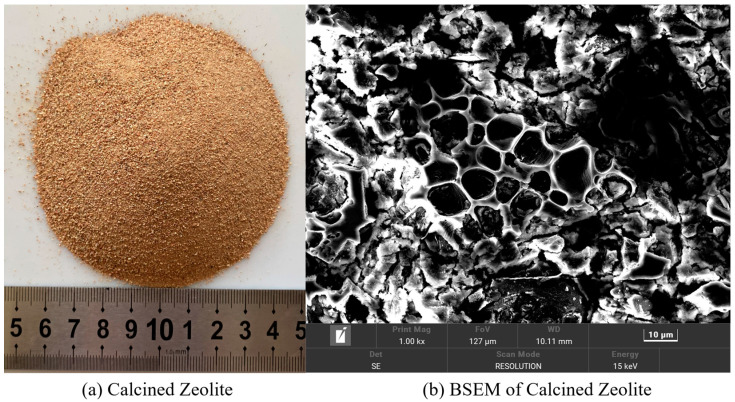
(**a**) Calcined zeolite, (**b**) backscatter plot of zeolite structure in matrix.

In this study, 52.5 ordinary silicate cement (OPC) was used in this test. F-type fly ash (FA), with smooth spherical surface particles (Figure 1a), was added to enhance the flowability of the fresh mixture. The nano-silica (SiO_2_) used had a SiO_2_ content of 99.8%, an average particle size of 40 nm, and a specific surface area of 200 m^2^/g. FACs are hollow, spherical particles with smooth surfaces produced by burning coal in power plants. FACs were characterized by smooth surface, hollow spherical particles with a particle size range of 0.01–0.3 μm and an average particle density of 530 kg/m^3^ (Figure 1b). Additionally, a standard polycarboxylic acid-based superplasticizer (SP) with a water reduction rate of 30% was employed to maintain a low water-to-cement ratio (W/B) of 0.2 in the LECC mixtures. Natural zeolites, possessing a significant number of framed pore structures, were used with an average particle size of 0.15 mm. The calcined zeolites and their internal pore structures after calcination for 30 min at 500 °C are depicted in Figure 2. The chemical compositions of the cement, fly ash, hollow beads, silica nanoparticles, and zeolites are presented in Table 1. To achieve high ductility and low-density ECC, polyethylene (PE) fibers were incorporated into the mixture, and the physical and mechanical properties of PE fibers are provided in Table 2.

### 2.2. Mixture Preparation

This experiment was conducted with a constant water-to-cement ratio (W/B) of 0.2 (by mass) to ensure consistent matrix strength of LECC. Different percentages (0%, 10%, 15%, and 20%) of pre-infiltrated zeolite were used to replace the cementitious materials (including cement, fly ash, and hollow spherical particles) in the LECC. The objective was to investigate the impact of a large dosage of zeolite on the self-crushing and mechanical properties of LECC. The specimens were named according to the amount of zeolite replacement, as shown in Table 3. For example, Z15 denotes a specimen with a 15% replacement of zeolite.

**Figure 3:**
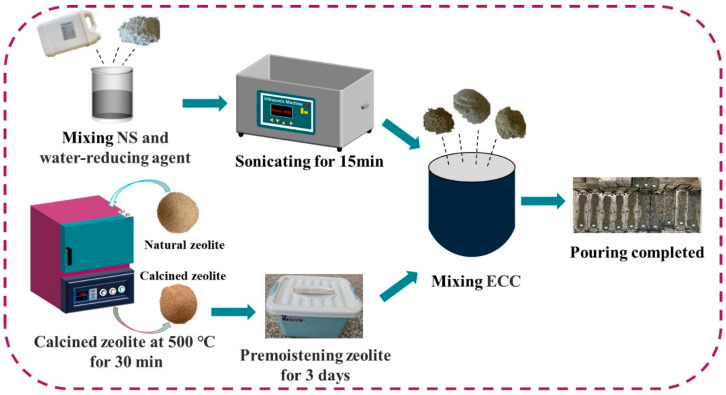
Flow chart of the experiment.

The experimental procedure is illustrated in Figure 3. In order to ensure uniform dispersion of the nano-silica (NS) in the LECC mixture, an ultrasonic dispersion pretreatment was employed. Firstly, the nano-silica was dissolved in water along with the water-reducing agent and thoroughly stirred using a glass rod. Subsequently, an ultrasonic machine with a power of 90 W was employed for 15 min of ultrasonication. Meanwhile, ordinary Portland cements (OPCs), fly ashes (FAs), and hollow beads (FACs) were added to the mixer and mixed at low speed for 2 min. Next, the pre-wetted zeolite and the aqueous solution of ultrasonically treated nano-silica were added, followed by a 2 min mixing at low speed and an additional 2 min mixing at high speed. Once the mixture attained consistent stability, polyethylene (PE) fibers were gradually added and mixed at high speed for 5 min. After achieving uniform fiber dispersion, the mixture was placed in a mold and vibrated for 2 min. In order to prevent moisture loss, the specimens were covered with cling film and kept indoors for 24 h before demolding. Subsequently, the samples were transferred to a standard conditioning room with a temperature of T = 20 ± 1 °C and a relative humidity of RH ≥ 98% for a curing period of 28 days.

### 2.3. Water Absorption of Zeolites

The water absorption ratio was determined following the methodology described in the literature [21,22]. Firstly, the calcined zeolite samples were submerged in water at a temperature of T = 20 ± 2 °C. After 48 h of submergence, the surface drying of the zeolite was performed, and the mass of the three samples was measured as *m*_0_. Subsequently, the samples were dried in an oven at 105 °C for 24 h, and the mass was measured as *m*_1_. The water absorption ratio of zeolite was calculated using Equation (1), and the experimental results were averaged. The water absorption of zeolite was determined to be 27.6%.

(1) $$W=\frac{m_{0}-m_{1}}{m_{0}}\times 100\%$$

where *W* is the water absorption ratio of the calcined zeolite at 48 h, *m*_0_ is the surface dry mass, and *m*_1_ is the dried mass.

### 2.4. Characterization of Physical and Mechanical Properties of LECC

#### 2.4.1. Bulk Density

Prior to conducting the compressive strength test, the compressed specimens underwent surface drying, and a mass test was performed to calculate the apparent density of the specimens.

#### 2.4.2. Compressive Strength 

For the test of compressive strength, three 70.7 mm × 70.7 mm × 70.7 mm cube specimens according to [23] were poured for each proportion, and after standard maintenance for 28 d, the results were tested using a universal testing machine, with the loading rate set at 0.6 MPa/s, and the results were averaged.

#### 2.4.3. Uniaxial Tensile Test

**Figure 4:**
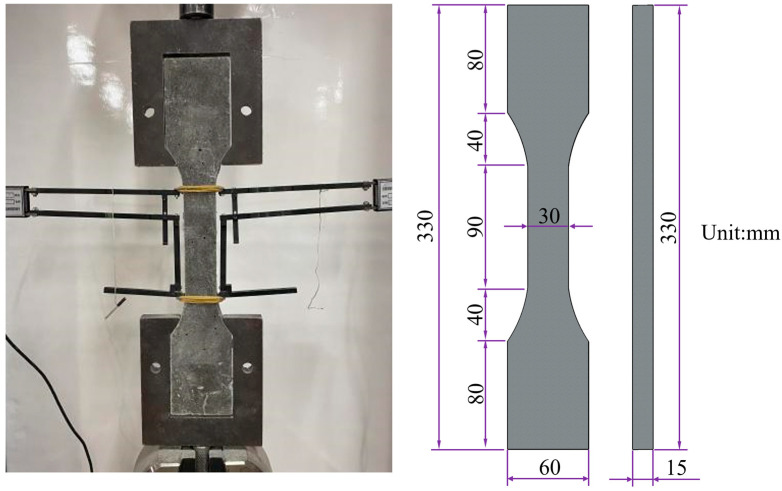
Dimensions of uniaxial tensile specimen and test device (unit: mm).

The tensile properties of LECC were tested by casting three dog bar bone specimens for each proportion; during the test, an extensometer was placed on both sides of the specimen, and the tensile properties were assessed by the uniaxial tensile test [9]. The shape and dimensions of the specimens are shown in Figure 4. The specimens were loaded by displacement at a loading rate of 0.5 mm/min. The results were averaged.

#### 2.4.4. Autogenous Shrinkage

**Figure 5:**
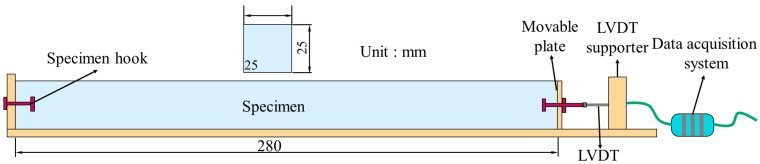
Autogenous shrinkage test device.

The autogenous shrinkage deformation was measured using a 25 × 25 × 280 mm^3^ elongated test mold [4], as illustrated in Figure 5. The test mold was wrapped with a double-layered plastic sheet. The homogenized LECC mixture was poured into a stainless steel pipe, which was also covered with a double-layered plastic sheet. A removable smooth polythene sheet was placed over the shrink tubing to minimize wall friction on the specimen. One end of the specimen was fixed to a steel spike, while the other end was fixed to a removable steel plate in contact with a measuring probe with an accuracy of ±1 μm for shrinkage measurements. The tests were conducted under isothermal sealed curing conditions, and the deformation values were recorded using a data logger. The entire shrinkage test was performed at a temperature of 20 ± 2 °C and a relative humidity of 50 ± 4% RH.

#### 2.4.5. Internal Relative Humidity

Before conducting the test, a PVA tube was prepared in advance with two small rectangular holes cut at one end to enable gas contact between the moisture sensor and the inside of the concrete. During the pouring process, the mixture was poured in order to make it more homogeneous. Firstly, 50 mm of LECC mixture was poured and vibrated for 1 min to homogenize the mixture at the bottom of the mold. Then, the PVA tube was positioned in the center of the sample, and the pouring process continued. In order to prevent the mixture from filling the space inside the tube, a plastic rod with an external diameter of 16 mm was inserted into the PVA tube during the pouring process.

The development of relative humidity (RH) within the concrete was monitored using a TH20BL-EX humidity sensor with an accuracy of ±2% RH. The specimens were placed inside the PVA tubes for humidity measurements 12 h after the completion of casting. The specimens used for humidity measurements were divided into two groups, each consisting of four specimens. One group of specimens was fully exposed to the test chamber environment after demolding, while the other group was completely sealed with cling film to prevent moisture exchange with the external environment. Measurements from the moisture sensors were taken at 10 min intervals, starting 12 h after the pouring process was completed.

### 2.5. Micro-Analyses

#### 2.5.1. Optical Electron Microscope

Tensile specimens with different zeolite doping were selected, crack widths were observed, and crack development was analyzed using scanning electron microscopy.

#### 2.5.2. SEM

The micro-morphology of the PE cross-section was examined using a scanning electron microscope (SEM). The SEM analysis involved observing the fracture morphology of the PE fibers, as well as the cross-section of the material. The SEM detector was operated at a low accelerating voltage of 15 kV to obtain detailed images.

## 3. Results

### 3.1. Physical and Mechanical Properties

#### 3.1.1. Bulk Density

**Figure 6:**
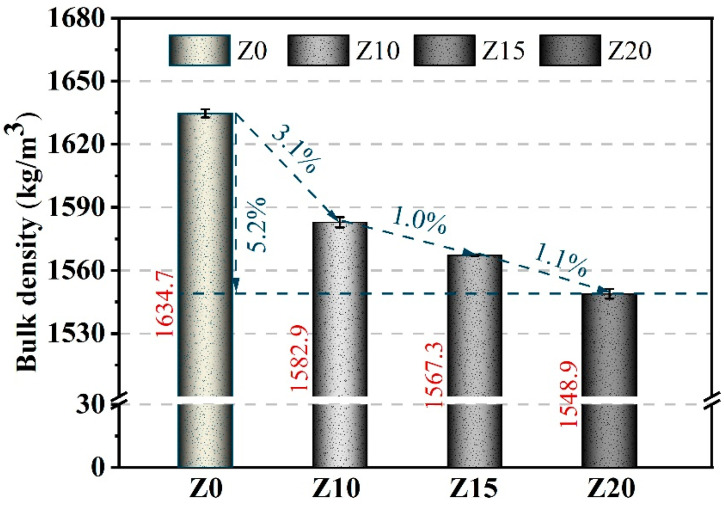
Bulk densities of LECC mixtures.

As depicted in Figure 6, the bulk density of LECC gradually decreased with the increasing dosage of zeolite. The density of all the samples ranged from 1550 to 1650 kg/m^3^. For instance, the apparent density of the LECC mixture with a zeolite admixture of 20% decreased to 1548.9 kg/m^3^ compared to the control group, representing a reduction of 5.2%. This significant decrease in density demonstrated the effective reduction in the LECC matrix density through the addition of zeolite. Moreover, according to the specifications outlined in JGJ 51-2002, the density of all LECC mixtures meets the requirements for lightweight concrete, i.e., a density below 1950 kg/m^3^.

#### 3.1.2. Compressive Properties

**Figure 7:**
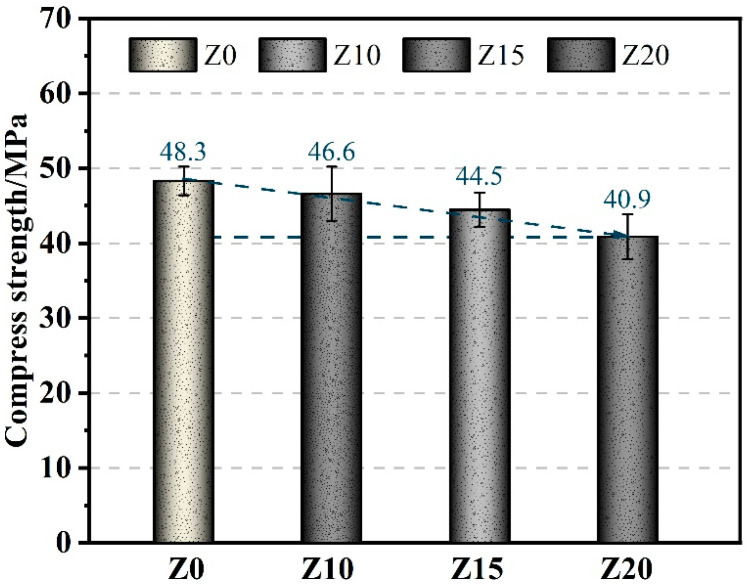
Compressive of LECC mixtures.

Figure 7 illustrates the average compressive strength test results of LECC at 28 days with different zeolite replacement amounts. The findings indicated that a high dosage of zeolite particles had a detrimental effect on the strength of the LECC matrix. As the zeolite replacement amount increases, the compressive strength of LECC consistently decreases. At a zeolite doping level of 10%, the loss of compressive strength was almost negligible, with only a 3.7% reduction compared to the control strength. At this dosage, the negative impact on strength caused by the porous framework structure of zeolite was counterbalanced by the positive effects of zeolite’s volcanic ash activity and internal curing water, resulting in similar compressive strength at the macroscopic level.

However, as the zeolite dosage continued to increase, the compressive strength decreased further. This can be attributed to two factors. Firstly, the reduction in cement and fly ash content by 20% compared to the control led to an increase in the actual water-to-cement ratio, thereby diminishing the matrix strength. Secondly, the replacement of a significant amount of zeolite with its porous structure introduced numerous internal pores, increasing the matrix porosity, and subsequently reducing the compressive strength. Interestingly, even at the maximum zeolite dosage of 20%, the compressive strength can still surpass 40 MPa, representing a decrease of only 15.3% compared to the control. This highlights the significant role of zeolite as an internal reservoir with volcanic ash properties in promoting cement hydration. Considering the sustained reduction of density discussed earlier, this indicated the potential to achieve low-density, high-strength cementitious materials.

#### 3.1.3. Tensile Properties

**Figure 8:**
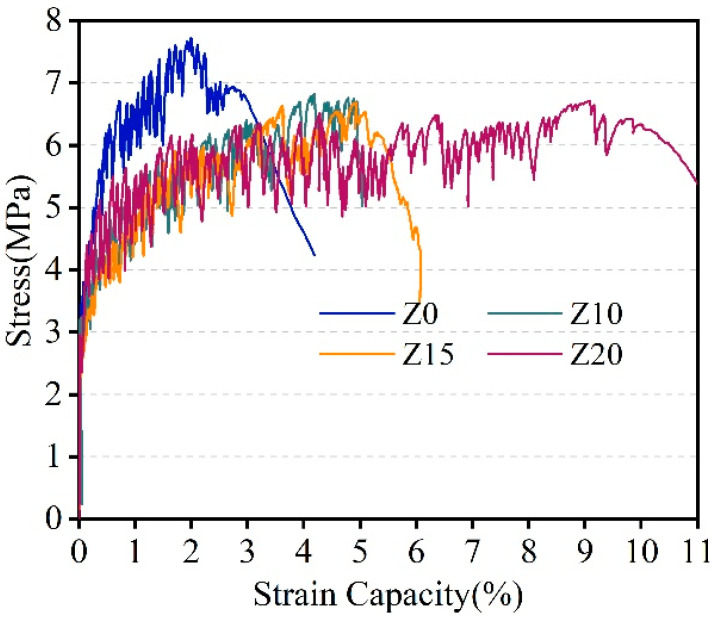
Tensile stress–strain curves of LECC mixtures.

Figure 8 presents the representative stress–strain curves of LECC with different replacement amounts of zeolite. The test results demonstrated excellent strain hardening and multi-seam cracking behavior in all specimens during the tensile process, which can be categorized into three stages:

A. Linear elastic rise stage: In the initial stage of the tensile process, the applied tensile stress is relatively small, and the stress–strain relationship follows a linear pattern. The slope of the linear curve represents the tensile modulus of elasticity, denoted as E. As the specimen develops cracks, the stress–strain relationship deviates from linearity. The point where the linear portion ends can be defined as the cracking point of the material. The corresponding tensile stress value at the cracking point represents the tensile initial cracking strength, and the corresponding strain value represents the tensile cracking strain.
B. Strain hardening and multi-seam cracking stage: After the occurrence of the first crack, additional cracks continue to form, leading to a reduction of crack width and a decrease in the tensile stiffness of the specimen. This process continues until the tensile stress reaches its peak value, which can be defined as the ultimate tensile strength. The strain value corresponding to the ultimate tensile strength is referred to as the ultimate tensile strain.
C. Strain softening stage: Once the peak stress is reached, new cracks generally cease to appear. The main crack forms and the crack width progressively increases. Consequently, the tensile stress gradually decreases until the specimen undergoes complete failure.

These stages illustrated the progressive damage process observed in the uniaxial tensile test of LECC specimens. The stress–strain curves captured in Figure 8 exemplify the strain hardening, multi-seam cracking, and strain softening behavior displayed by LECC samples with different zeolite replacement amounts.

**Figure 9:**
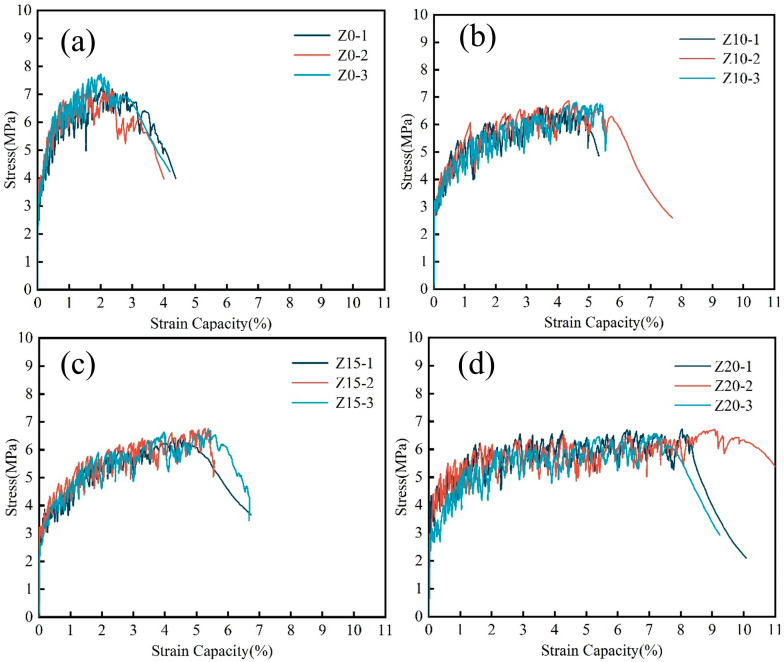
Tensile stress–strain curves of LECC mixtures. (**a**–**d**) represent the three stress-strain curves for Z0, Z10, Z15, and Z20 respectively.

Figure 9 shows the tensile stress–strain curves of LECC in the past experiment. The test results demonstrated that all specimens exhibited excellent strain hardening and multi-seam cracking, with ultimate tensile properties ranging from 2.1% to 8.1%. These values were significantly higher, hundreds of times greater, than those typically observed in ordinary concrete. In particular, when the zeolite replacement reached 20%, the ultimate tensile strain exceeded 8% and reached 8.1%, which represented a significant increase of 277.7% over the control group. This substantial improvement greatly enhanced the ductility of the matrix and provided the possibility of achieving lightweight, high-strength, and high-toughness properties in the material.

**Figure 10:**
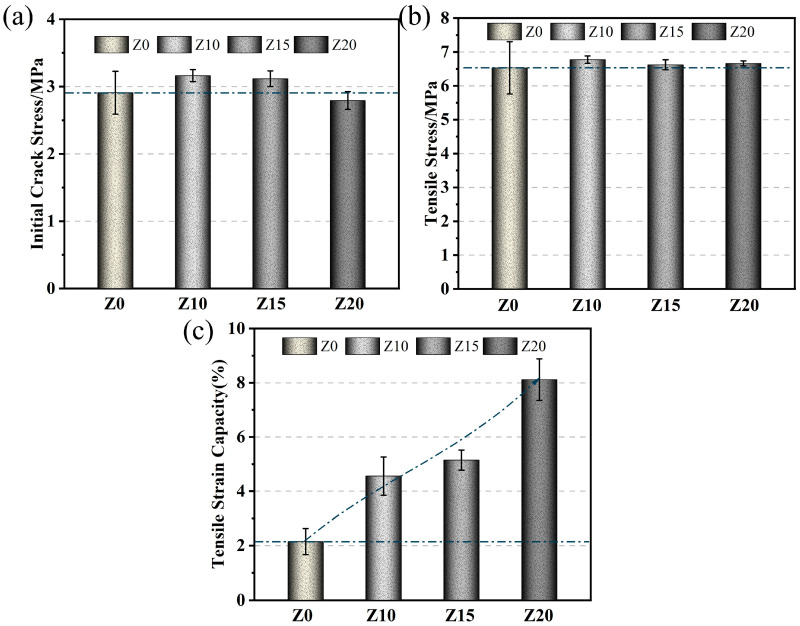
Tensile performances of LECC mixtures. (**a**) Initial crack strength; (**b**) Ultimate tensile strength; (**c**) Ultimate tensile strain.

The tensile property index was an important parameter for ECC materials. Three parameters that quantify the tensile properties (including initial crack strength, ultimate tensile strength, and ultimate tensile strain) are presented in Figure 10. The initial crack strength and ultimate tensile strength of LECC increased and then decreased with the increase in zeolite substitution. The ultimate tensile strength was almost flat for different zeolite replacement amounts (Z10, Z15, and Z20). This is because the addition of zeolite reduces the LECC matrix strength. With the significant increase in ultimate tensile strain, the tendency of decreasing ultimate tensile strength was alleviated.

#### 3.1.4. Crack Pattern Development to Explain Tensile Properties

The limiting mean crack width was used as an index to evaluate the crack control capability of the material. During the tensile strain hardening stage of the material, the sum of the opening widths of all cracks and the elastic deformation of the uncracked region of the material was measured as the total tensile deformation using LVDTs. Then, the total crack width *W*_total_ [24] was calculated:

(2) $$W_{\mathrm{total}}=L(\varepsilon -\sigma /E)$$

where *L* is the LVDT scale length, *ε* is the measured tensile strain, *σ* is the measured tensile stress, and *E* is the modulus of elasticity of the material. When the tensile stress reaches the ultimate tensile strength *σ_t_*, the final average crack width *W* of the material can be calculated by counting the number of cracks within the LVDT scale length, which can be expressed as

(3) $$W_{\mathrm{ave}}=L(\varepsilon_{t}-\sigma_{t}/E)/N$$

where *N* is the number of cracks and is the average of the number of cracks on both sides of the specimen.

**Figure 11:**
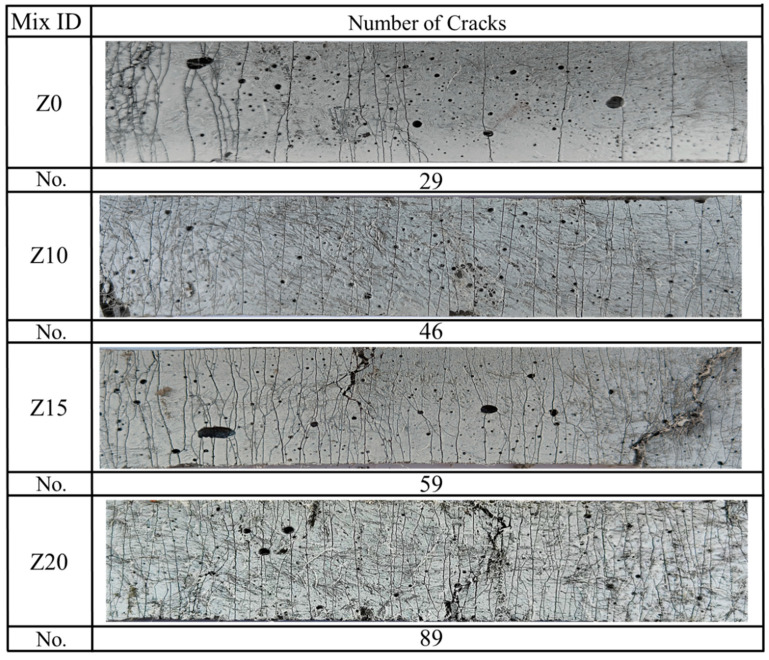
Crack pattern of LECC.

Figure 11 represents the representative crack patterns of the 100 mm rectangular portion of the ECC at failure, and all the LECC specimens showed multiple microcracks, with the zeolite-substituted matrix all having more cracks than the control. Moreover, there was a clear trend that more cracks smaller than 100 μm were observed with increasing zeolite doping, with a slight increase in the number of cracks with widths greater than 140 μm. For example, the cracks of Z20 were mainly distributed between 60 and 100 μm, with 32 cracks at 60–100 μm. In contrast, most of the cracks in Z0 were in the range of 80–120 μm. This is due to the doping of zeolite, which brings higher porosity and optimizes the multi-seam cracking characteristics of LECC [25].

**Figure 12:**
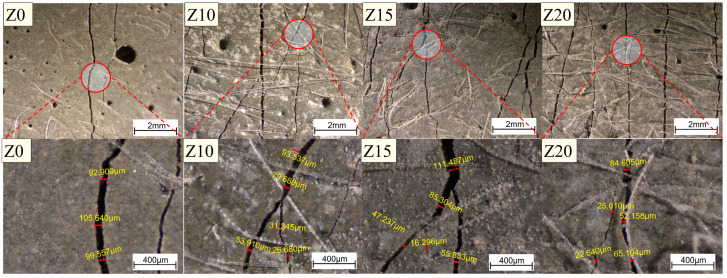
Optical microscope view of crack development. Red circles are zoomed in areas.

Figure 12 shows the details of crack development. From the figure, it can be found that the cracks in the control group were generally through cracks and rarely found to have extended cracks; while with the increase in zeolite replacement, the extended cracks gradually appeared, and the width of the extended cracks gradually decreased with the increase in zeolite doping.

**Figure 13:**
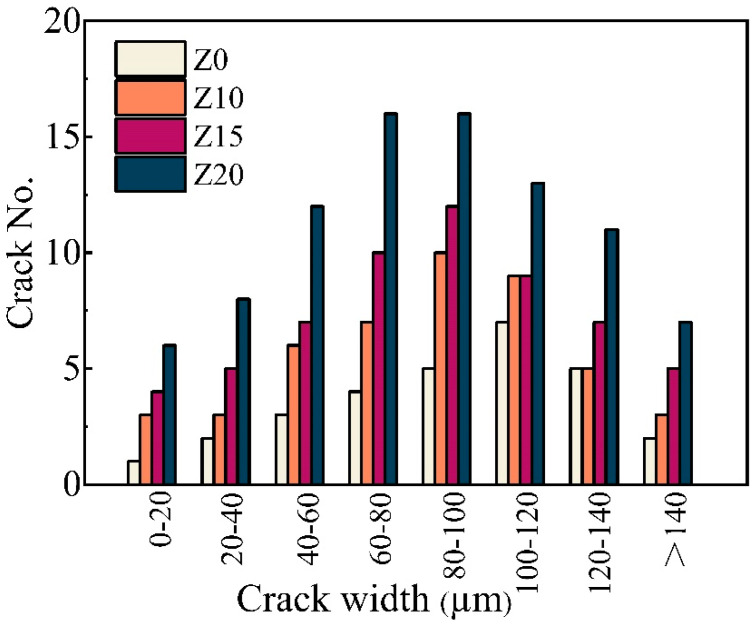
Distribution of crack numbers for different ECCs at specimen failure.

Figure 13 summarizes the crack number as well as the average crack width distribution. The number of cracks in the LECC specimens increased from 29 to 89 with the increase in zeolite doping, and the average crack width gradually decreased with the increase in zeolite doping. The average crack widths of Z0, Z10, Z15, and Z20 were 121 μm, 102 μm, 98 μm, and 91 μm, respectively. The zeolite substitution of 20% exhibited the minimum average crack width of 91 μm, which was lower than that of the control by 24.8%.

### 3.2. Shrinkage Resistance

**Figure 14:**
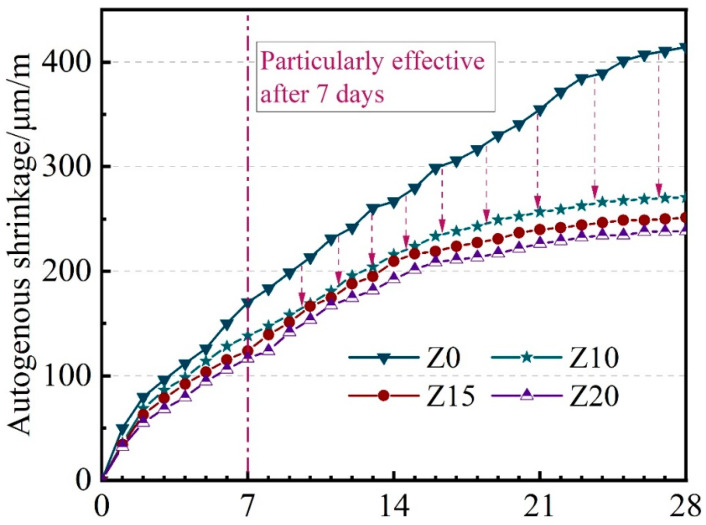
Autogenous shrinkage development of LECC containing various replacement.

As shown in Figure 14 from the past experiment, a large dosage of zeolite substitution was found to significantly reduce the autogenous shrinkage of LECC. The autogenous shrinkage of LECC decreased continuously as the proportion of zeolite replacement increased. When the zeolite substitution reached 20%, the autogenous shrinkage of the LECC matrix was significantly alleviated compared to the control group.

This reduction of autogenous shrinkage can be attributed to several factors. Firstly, the zeolite, which carried moisture, entered the interior of the matrix and provided a constant source of moisture for cement hydration. This resulted in more complete cement hydration and the formation of calcite and other cementitious products, which generated expansion pressure [26,27]. These expansion pressures partially counteracted the self-contraction of LECC, thus reducing its autogenous shrinkage.

Additionally, as the zeolite ages, it developed a fixed porous framework structure, which also exerted an inhibitory effect on the contraction of the LECC matrix during the later stages of curing. This effect slowed down the self-contraction of LECC, further contributing to the reduction of autogenous shrinkage over time.

### 3.3. Internal Humidity of the Specimens

**Figure 15:**
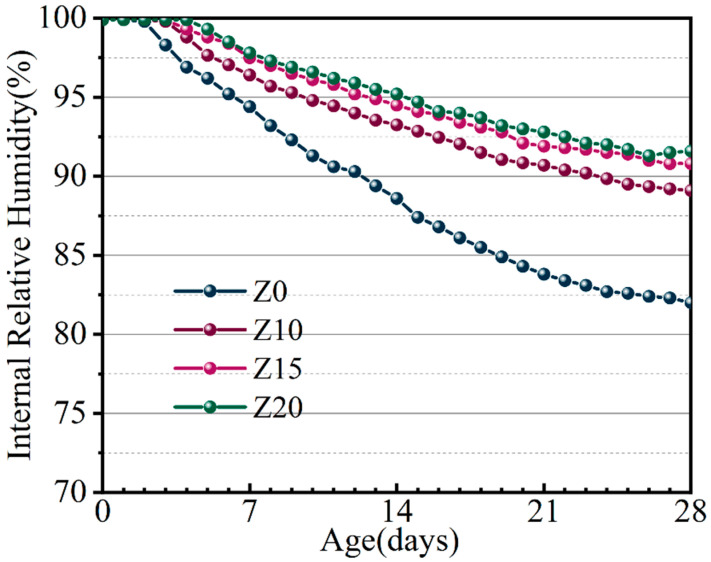
Humidity development inside the specimen.

Jun Zhang et al. [28] proposed a two-stage model to describe the development of humidity in the center of the LECC specimen: the water vapor saturation stage (Stage I) at 100% relative humidity (RH) and the stage of gradual RH decrease (Stage II). The development of relative humidity inside the specimen effectively reflects the shrinkage regulation principle of LECC (Figure 15).

During the conditioning process, the ratio of internal RH reduction decreases as the zeolite replacement amount increases. When the zeolite replacement amount reached 20%, the internal RH of LECC experienced the most significant relief, reaching 85.6%. Compared to the control group, the internal RH increased by 16.2% after 28 days. The zeolite played a crucial role in significantly increasing the internal RH of the specimen, which, in turn, promoted more adequate hydration reactions.

## 4. Discussion

### 4.1. Mechanism of Bridging Properties of PE Fibers

**Figure 16:**
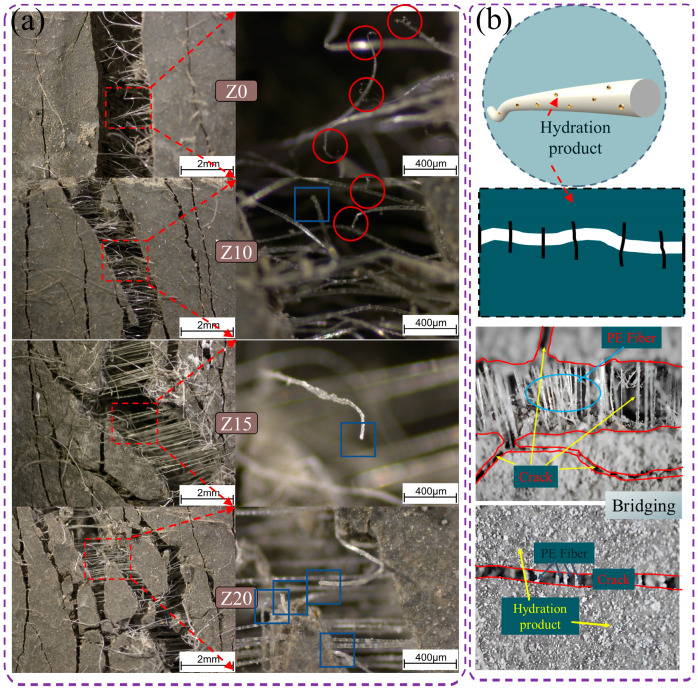
The PE fiber tension diagram of the specimen after stretching. (**a**) The red circle indicated that the PE fiber was ruptured and the blue box indicated that the PE fiber was pulled out; (**b**) state of fiber tension.

Figure 16a was the fiber tension diagram of the specimen after stretching, and Figure 16b was the fiber tension diagram. As shown in Figure 16a, polyethylene fibers were well dispersed in LECC, can withstand excellent tensile loads, and play an important role in fiber bridging in LECC. As shown in Figure 16a, PE fibers were well dispersed in LECC, can withstand excellent tensile loads, and played an important role in fiber bridging in LECC. Li et al. [29] pointed out that an optimum range of frictional stress existed to obtain steady-state crack propagation at a certain matrix toughness. When the frictional stress is smaller than the minimum value, fiber pull-out facilitates, whereas fiber rupture or premature failure happens when the frictional stress is larger than the maximum value. According to the morphology of PE fiber, with the increase in zeolite content, the frictional stress was lower than that of the control sample, and the fiber fracture rate may decrease, resulting in the gradual decrease in the possibility of PE fiber breaking. In addition, in the left column of Figure 16a, it can be seen that the observed fiber tension length significantly improved with the increase in zeolite replacement. At Z0, most PE fibers were in a broken state, and with the increase in zeolite content, the length of PE fibers stretched outside the matrix increased significantly. As a result, the pull-out mode of the fibers gradually changed from rupture to pull-out, which increased the chance of fiber pull-out and improved the ductility performance. This greatly increased the ultimate tensile strain of LECC.

**Figure 17:**
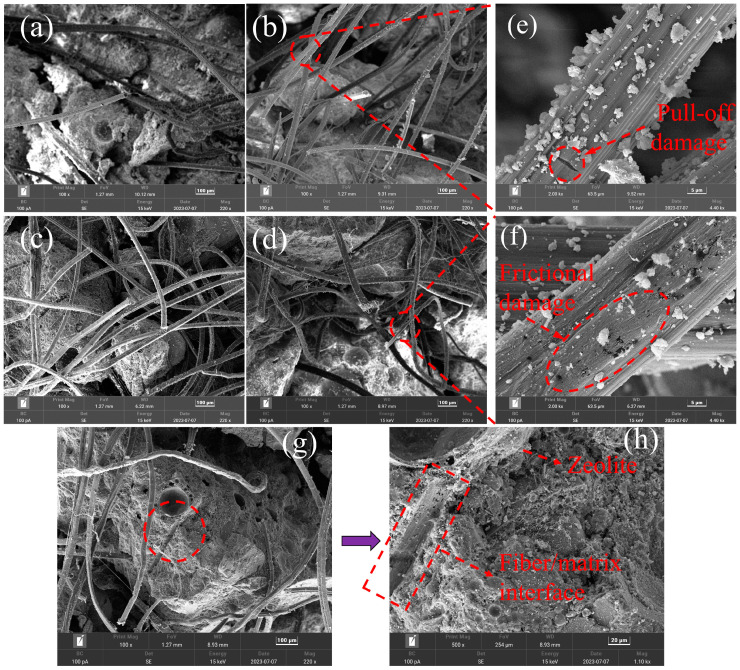
SEM images of PE fiber fracture patterns. (**a**–**d**) represent Z0, Z10, Z15, and Z20, respectively. (**e**,**f**) SEM images of PE fiber pull-out modes for Z10 andZ20, respectively. (**g**,**h**) SEM image of fiber-matrix interface.

In studying the interfacial bonding properties between fibers and matrix, the preparation of small samples from slices of the stretched dog bone specimen and the pull-out surface of the fibers were analyzed with a scanning electron microscope. As shown in Figure 17, polyethylene (PE) fibers were deformed during the pull-out process. This meant that the mechanical interactions occurring between the fibers and the matrix contributed to wasted energy and increased mechanical friction. This is due to the fact that zeolite is a lightweight porous structure with low strength compared to cement paste; therefore, the addition of zeolite will reduce the fracture strength of the matrix, whereas low fracture toughness will be beneficial for the formation of multi-seam cracking in terms of the design principle of ECC [30]. From Figure 17e,f, it can be observed that the fiber morphology gradually changed from surface cracking to surface friction damage with the increase in zeolite doping. This indicated that the interfacial bond strength between the fiber and the matrix gradually decreased, which was also with the increase in zeolite doping; the ultimate tensile strain was as high as 8.1% when the zeolite doping was 20%, the reason for the significant increase in ultimate tensile strain. In particular, the ultimate tensile strain was as high as 8.1% when the zeolite doping was 20%. This was the main reason for the gradual increase in the number of cracks from 39 to 89, the significant increase in the number of cracks as well as the substantial increase in ductility.

Figure 17g,f shows an SEM image of the fiber-matrix interface. It was clearly observed that zeolite belonged to a porous structure. Zeolite doped into the matrix as an external dopant made the matrix toughness decrease, and the bond strength of PE fibers to the matrix decreased. PE fibers were pulled out from the matrix when they were tensile and formed a complete fiber channel.

### 4.2. Analysis of Zeolite Autogenous Shrinkage Mitigation Mechanism

Based on good mechanical and thermodynamic studies [31], usually due to loss of water during hydration or drying of cement, a meniscus may be formed, causing an increase in capillary pressure, which in turn causes shrinkage of the cementitious material [32,33]. As the age of maintenance progresses, the cement gradually hydrates, the free water in the matrix gradually decreases, and the internal relative humidity decreases; therefore, a large number of pores are formed in the hardened cement paste, and the water saturation in the pores decreases. As the saturation state of capillary pores changes from saturated to unsaturated, the concave surface within the pores is subjected to internal pressure. In order to keep the concave surface in equilibrium, the capillary tension increases, and thus autogenous shrinkage occurs [34].

**Figure 18:**
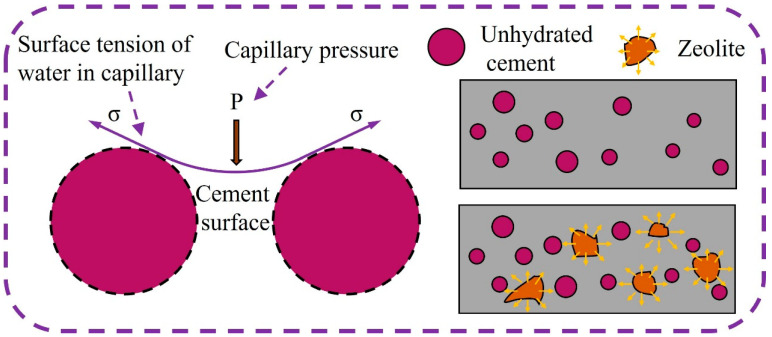
Schematic diagram of capillary water tension.

On the one hand, with the increase in zeolite replacement, the internal conservation water carried by zeolite continuously released free water during cement hydration to provide sufficient water for cement hydration, and the capillary water in the hardened cement paste was supplemented or even saturated, and the concave surface in the pores was subjected to reduced pressure, and the capillary tension was relieved, which was macroscopically manifested as the relief of autogenous shrinkage. On the other hand, the rigid framework structure and high modulus of elasticity of zeolite helped to inhibit the deformation of autogenous shrinkage [35], as shown in Figure 18.

### 4.3. Mechanism of Zeolite Enhancement of LECC Ductile Properties

**Figure 19:**
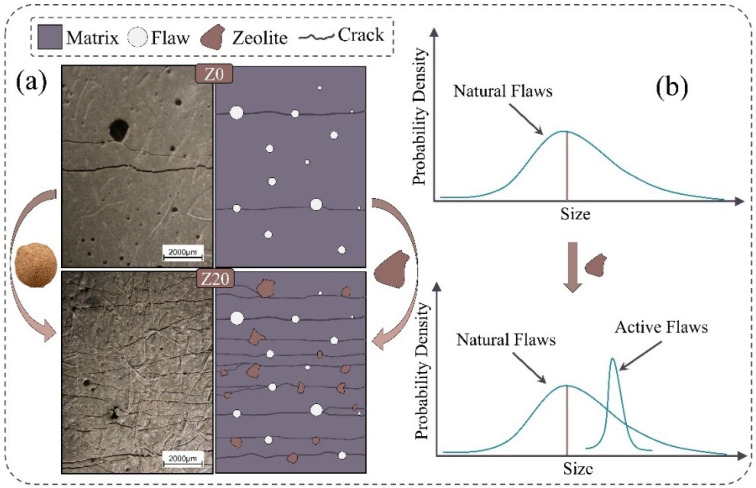
Mechanism of LECC ductility enhancement after zeolite replacement (**a**,**b**). The use of zeolite tunes the distribution of defects in the high-strength matrix, leading to excellent strain hardening properties of LECC.

Figure 19 summarizes the enhancement mechanism of zeolite incorporation on the ductility properties of LECC. The incorporation was able to significantly enhance the ductility properties of the LECC matrix. The enhancement of ductility performance can be explained by two reasons. One of them was that the porous structure of zeolite becomes active defects in LECC, and according to the ECC design theory, the higher the probability of active defects, the more prone to multiple cracking phenomena [36,37]. In addition, zeolite, as a material with a porous framework structure, reduced the matrix strength after doping into LECC mixtures, changing the mode of polyethylene fiber-to-interfacial bonding property damage from pull-off to pull-out, which implied that the matrix has a relatively higher deformation capacity, and when the LECC was subjected to tensile pull-outs, the zeolite material can absorb and dissipate the strain energy through deformation, which improved the material’s ductility performance.

## 5. Conclusions

In this study, the effect of massive zeolite replacement on the mechanical properties of LECC, especially the tensile properties, as well as the autogenous shrinkage properties, was evaluated. Based on the experimental results as well as microstructural analyses, the following conclusions can be drawn:

1. The addition of a large dosage of zeolite can significantly reduce the apparent density of LECC. With the increase in zeolite doping, the density of LECC decreased gradually. When the zeolite replacement amount reached 20%, the density was significantly reduced by 5.2% compared to the control, further reducing the apparent density of LECC.
2. With the addition of zeolite, although the number of large cracks increased and the compressive strength was slightly reduced, the average crack width of LECC decreased somewhat, and the ultimate tensile strain capacity was significantly increased.
3. Due to the increase in active defects and the decrease in bonding properties at the fiber-matrix interface, zeolite with a porous framework structure can significantly increase the strain hardening capacity of LECC, the tensile mode was changed from fiber pull-off to fiber friction pull-out, and the surface of PE fiber pull-out process was severely damaged.
4. The addition of pre-infiltrated calcined zeolite can well alleviate the autogenous shrinkage of the LECC matrix. With the increase in zeolite replacement, the autogenous shrinkage of all LECC specimens was alleviated. In addition, the addition of zeolite alleviated the autogenous shrinkage of LECC at the later stage, most significantly.

Higher ductility and lower density are more advantageous in the reinforcement of building structures. Therefore, for approximately the same ultimate tensile strength, the zeolite replacement of 20 percent was optimal as it has less autogenous shrinkage and higher ductility.

## Figures and Tables

**Table 1:** Chemical composition and physical properties of cement, FA, NS, FACs, and zeolite.

Chemical Analysis	CaO	Fe_2_O_3_	MgO	SO_3_	K_2_O	SiO_2_	Na_2_O	Al_2_O_3_	TiO_2_	Others
OPC	65.52	2.95	3.24	3.84	0.41	17.51	0.42	4.22	1.28	0.61
FA	3.30	8.09	1.34	0.67	1.37	53.00	0.34	24.19	-	7.7
FACs	1.06	1.96	-	0.42	3.94	73.10	2.42	16.70	0.35	0.05
SiO_2_	-	-	-	-	-	99.80	-	-	-	0.20
Zeolite	4.22	3.54	1.72	0.13	3.06	72.07	0.67	13.35	0.57	0.67

**Table 2:** Physical and mechanical properties of PE fibers.

	Length/mm	Diameter/ μm	Fiber Strength/MPa	Modulus of Elasticity/GPa	Specific Gravity/g/cm^3^
PE fiber	12	26	2900	116	0.97

**Table 3:** Compounding ratios for LECC mixtures (kg/m^3^).

Mixture No.	C	FA	NS	Zeolite	FACs	Water	SP	PE Fiber (%)
Z0	874.0	391.5	39	-	195.8	251.7	101.7	1.75
Z10	786.6	352.4	39	146.1	176.2	251.7	101.7	1.75
Z15	742.9	332.8	39	219.2	166.4	251.7	101.7	1.75
Z20	699.2	313.2	39	292.3	156.6	251.7	101.7	1.75

## Data Availability

The authors declare that they have no known competing financial interests or personal relationships that could have influenced the work reported in this paper.
